# A new Bi-based visible-light-sensitive photocatalyst BiLa_1.4_Ca_0.6_O_4.2_: crystal structure, optical property and photocatalytic activity

**DOI:** 10.1038/srep23235

**Published:** 2016-03-17

**Authors:** WenWu Zhong, YanFang Lou, ShiFeng Jin, WenJun Wang, LiWei Guo

**Affiliations:** 1 Department of Materials, Taizhou University, Taizhou 318000, China; 2 Research & Development Center for Functional Crystals, Beijing National Laboratory for Condensed Matter Physics, Institute of Physics, Chinese Academy of Sciences, Beijing 100190, China

## Abstract

A new compound of BiLa_1.4_Ca_0.6_O_4.2_ is synthesized through solid state reaction, where the Ca substitutes, in part, the La site in a stable BiLa_2_O_4.5_ phase. The structure of the BiLa_1.4_Ca_0.6_O_4.2_ crystallizes in space group R3mH with a hexagonal lattice constants of a = 3.893(1) Å, c = 9.891(1) Å. Its optical absorption edge is about 2.05 eV, which just spans the visible light region. The photocatalytic activity of the BiLa_1.4_Ca_0.6_O_4.2_ powder to degradation of RhB under visible light irradiation is measured and improved more than 7 times by annealing in nitrogen ambient, indicating that annealing in nitrogen can effectively improve the photocatalytic activity by producing oxygen vacancy. Although the absolute photocatalytic activity obtained is low, there is great potential for enhancing the activity such as nanoscaling, doping, and coupling with other compounds.

With the aggravation of environmental pollution from hazardous organic compounds, more attention has been attracted to develop new photocatalytic materials for degradation of organic pollutant^1^,^2^,^3^. Metal oxides such as TiO_2_, ZnO, Bi_2_O_3_, and BiOCl^4^,^5^,^6^,^7^,^8^ are widely studied due to their potential in photocatatic applications. However, these metal oxides possess a wide band gap, which prohibits the effective utilization of the solar spectrum. Therefore, exploring new compounds with band gaps in visible light wavelength range is desirable. Among these compounds, bismuth-based oxides are known to exhibit rich structural diversity and high efficiency in degradation of organic pollutants, such as BiFeO_3_^9^, Bi_2_WO_6_^10^,^11^,^12^, BiPO_4_^13^,^14^, BiVO_4_^15^, BiOI^16^,^17^,^18^,^19^, and BiPbO_2_Cl^20^. The bismuth-based oxides possess hybridized band structure, which not only decreases the effective masses of electrons and holes effectively enhancing carrier transportation, but also narrows band gap extending light absorption to longer wavelength region^21^. BiLa_2_O_4.5_^22^, a compound possessing a monoclinic unit cell with superstructure, shows a yellow color suggesting its potential as visible light effective photocatalyst. However, our early experiments confirmed that its photocatalytic activity was poor compared with the above mentioned Bi-based oxides^13^,^14^,^15^,^16^.

To improve photocatalytic activity of a catalyst, various methods are used^23^,^24^,^25^, including the defect engineering^26^. Introduction of defects in a catalyst can boost interfacial charge transfer, thus effectively reducing recombination rate of photo-generated electrons-holes^26^. Pei *et al.*^27^ revealed that higher defect concentration in ZnO enhanced photogenerated charge carriers separation and transportation, resulting in an enhanced photocatalytic activity. Kong *et al.*^28^ revealed that tuning the defect concentration in TiO_2_ leads to high photocatalytic activities. Many other methods like high temperature calcination^29^, cold plasma treatment^30^, annealing in atmosphere^31^, and particle bombardment^32^ had been reported for creating defects in a catalyst. Compared with these methods, chemical doping is a simple and controllable way to adjust defects state and its concentration.

Herein, Ca is chosen to dope into BiLa_2_O_4.5_ to modify its structure, properties and even its photocatalytic performance. A new Bi-based compound BiLa_1.4_Ca_0.6_O_4.2_ is successfully synthesized. Its crystal structure, some optical properties and its photocatalytic activity are studied. It is found that the structure of BiLa_2_O_4.5_ changes from monoclinic to rhombohedral by doping of Ca. The surface oxygen vacancy concentration of the sample is modulated by annealing in a nitrogen ambient. The photocatalytic activitie of sample is increased by annealing in nitrogen.

## Results

**Figure 1:**
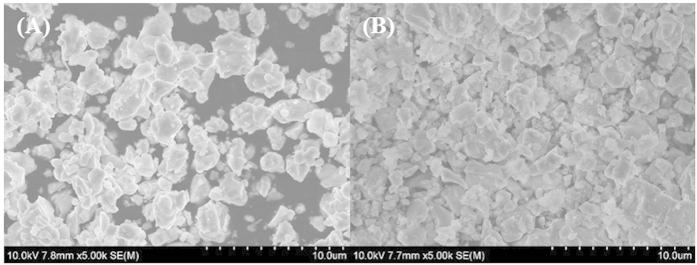
SEM images of BiLa_1.4_Ca_0.6_O_4.2_ (**A**) and annealed in nitrogen (**B**).

In order to analyze the morphology and crystallite size, SEM was adopted for studying the BiLa_1.4_Ca_0.6_O_4.2_ without and with annealing in a nitrogen ambient. From Fig. 1, it could be seen that there is no discernible change in the morphologies of samples with/without annealing in nitrogen ambient, and the average crystallite size is about 2 μm.

**Figure 2:**
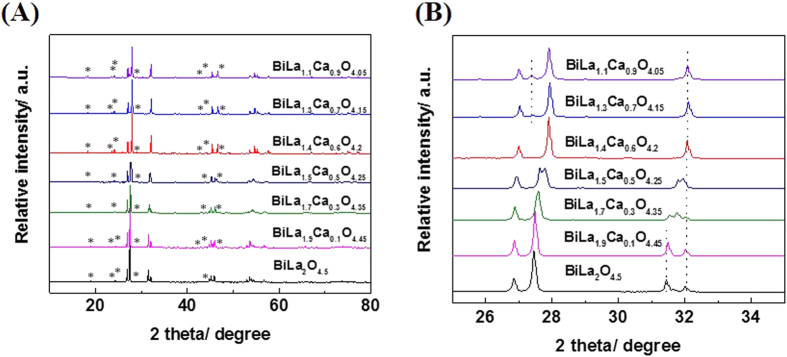
(**A**) X-ray powder diffraction patterns of BiLa_2−x_Ca_x_O_4.5−δ_, peaks marked by * are due to superstructure lines. (**B**) Partial enlarged drawing of (**A**).

Figure 2(A) shows the data of the X-ray powder diffraction pattern of the samples doping the BiLa_2_O_4.5_ with different Ca content, and Fig. 2(B) is the partial enlarged drawing of Fig. 2(A). From Fig. 2(B), with the increase of Ca content, the peak at 27.5° gradually shifts to higher angle, and the peak at 31.5° gradually reduces and disappears. The structure of BiLa_2−x_Ca_x_O_4.5−δ_ changes from monoclinic C2/m to rhombohedral R3mh with the increase of x value. A pure phase of BiLa_1.4_Ca_0.6_O_4.2_ is obtained when x = 0.6 with a rhombohedral R3mh structure. The peak at 27.3° is emerged when x > 0.6, which means that the sample has emerged a new impure phase. The main diffraction peaks of BiLa_1.4_Ca_0.6_O_4.2_ can be indexed using a rhombohedral cell with hexagonal lattice parameters a = 3.893(1) Å, c = 9.891(1) Å. The main diffraction peaks of BiLa_2_O_4.5_ can be indexed using a monoclinic cell with lattice parameters a = 6.8272(6) Å, b = 3.9885(2) Å, c = 4.0523(5) Å, which is consistent with the literature^22^,^33^. The peaks marked by ^*^ belongs to superstructure^33^,^34^,^35^,^36^,^37^, and the hexagonal lattice parameters are a = 31.144 (8) Å, c = 19.782 (2) Å.

**Figure 3:**
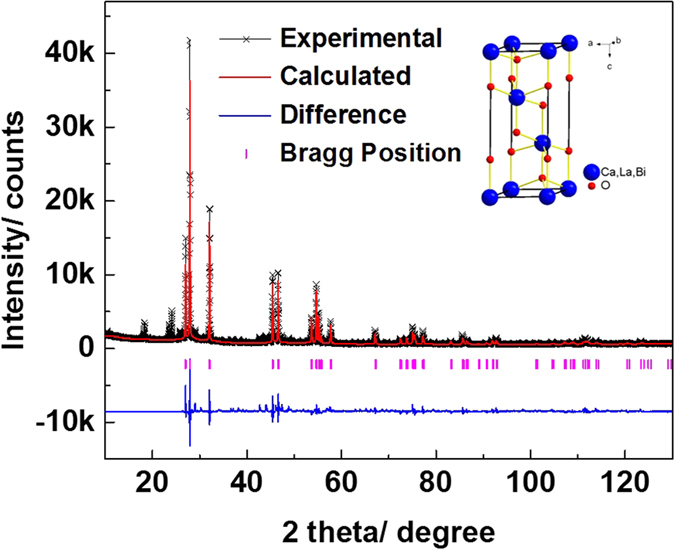
Rietveld refinement on powder XRD diffraction data of the BiLa_1.4_Ca_0.6_O_4.2_ sample. The black fork stands for experimental data, the red solid lines for calculated results and the blue solid lines at the bottom for the difference between the experiment and the calculation, the pink vertical bars indicate Bragg diffraction peak positions. The inset shows the crystal structure of rhombohedral BiLa_1.4_Ca_0.6_O_4.2_.

The structure of BiLa_1.4_Ca_0.6_O_4.2_ is then refined through Rietveld refinement on the diffraction data. The simulated result is shown in Fig. 3. The final agreement factors converge to *R*_*p*_ = 9.66%, *R*_*wp*_ = 12.86%, and *R*_*exp*_ = 3.29%, validating the reliability of our results. The refined lattice parameters are a = 3.893(1) Å, c = 9.891(1) Å for the hexagonal cell, which are consistent with that reported in the literature^33^. The inset of Fig. 3 shows the crystal structure deduced from the refinement, and the Bi, La and Ca atoms are randomly occupied the atomic sites marked with the bright blue balls.

**Figure 4:**
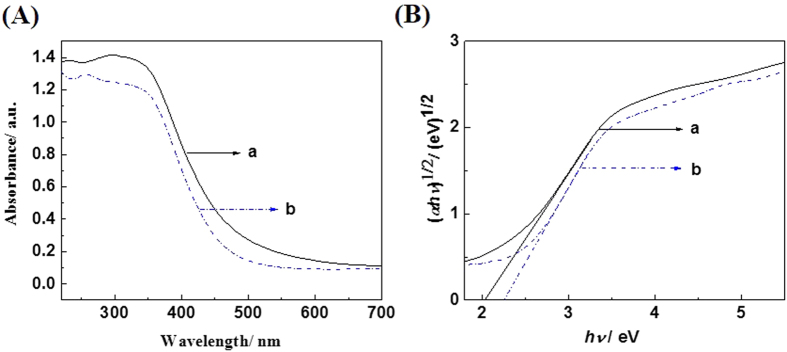
(**A**) UV-vis diffuse reflectance spectra and (**B**) plots of (*αhν*)^1/2^ versus the photon energy (*hν*) of the BiLa_1.4_Ca_0.6_O_4.2_ sample. The curves of a and b are from the as-prepared samples and the samples annealed in nitrogen ambient.

Figure 4(A) shows the UV-vis diffuse reflectance spectra of the prepared BiLa_1.4_Ca_0.6_O_4.2_ samples and samples annealed in nitrogen ambient, respectively. As shown in Fig. 4(A), the absorption edges of the BiLa_1.4_Ca_0.6_O_4.2_ samples without and with annealed in nitrogen ambient are at about 490 nm and 475 nm respectively. The absorption edge is blue-shifted after annealing in nitrogen ambient. Figure 4(B) shows the plots of (*αhν*)^1/2^ versus the photon energy (*hν*), An indirect band gap is deduced for the BiLa_1.4_Ca_0.6_O_4.2_, and a band gap about 2.05 eV (2.25 eV) deduced for the as prepared (nitrogen annealed) BiLa_1.4_Ca_0.6_O_4.2_ sample.

## Discussion

**Figure 5:**
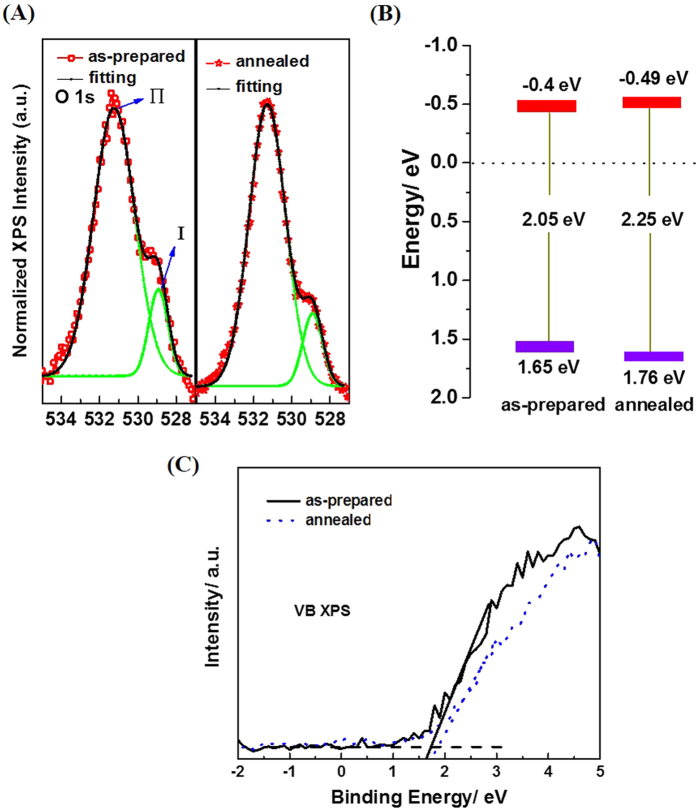
Normalized O 1 s XPS spectra (**A**), the band edge energy diagram (**B**) and the Valence-band XPS spectra of the samples (**C**). In (**B**) the red and the purple marks represent the conduction and valence band edges respectively.

Figure 5(A) shows the O 1*s* peaks of two types of the samples (i.e. as-prepared sample and the sample annealed in nitrogen). The O 1*s* state splits into two peaks, Ι and Π, located at 528.6 eV and 531.2 eV, respectively. The peak Ι can be attributed to the lattice oxygen in the BiLa_1.4_Ca_0.6_O_4.2_, while the peak Π can be due to the adsorbed oxygen in the surface of the sample, such as O^−^ or OH^−^ 38,39,40. The strong peak Π in the two types of samples could be ascribed to that the samples are composed of the micrometer-sized BiLa_1.4_Ca_0.6_O_4.2_ particles, whose specific surface area is high corresponding to a large bulk single crystal. The peak intensity ratio of Π/Ι are about 6.58, 6.95 for the as-prepared samples and the samples annealed in nitrogen ambient, respectively, indicating that the amount of adsorbed oxygen increases as more oxygen vacancies are created^41^.

The density of states (DOS) of the valence band of samples was also measured by valence band XPS (Fig. 5(C)). The valence bands (VB) are about 1.65 and 1.76 eV relative to the standard hydrogen electrode (SHE) for the samples prepared and annealed in nitrogen, respectively. Combined with the results from optical measurement, we can conclude that the conduction band (CB) minimum would occur at about −0.4 and −0.49 eV for the samples prepared and the annealed in nitrogen, respectively.

According to the UV-vis diffuse reflectance spectra and the valence-band XPS spectra, energy band edge diagram of the BiLa_1.4_Ca_0.6_O_4.2_ is drawn as shown in Fig. 5(B). As illustrated in Fig. 5(B), the band edges of the CB and VB of BiLa_1.4_Ca_0.6_O_4.2_ just well^42^ stride the reduction H^+^/H_2_O and oxidization O^2−^/H_2_O electrodes, favoring the electron injection from the photocatalyst to the dye, which is beneficial to play reduction action and degrade the dye molecule. The little broaden band gap of the annealed sample in nitrogen ambient is probably due to the forming of nitride-oxygen vacancy complexes which change the CB and the VB edges as model calculated by others^43^ and an experimental observation in a Cu_2_O film^44^.

**Figure 6:**
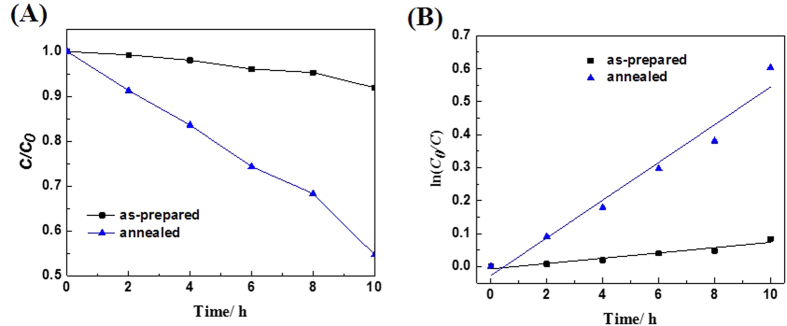
(**A**) Photocatalytic activites of BiLa_1.4_Ca_0.6_O_4.2_ under visible light, (**B**) The first order Kinetics of degradation of RhB in solution.

Figure 6(A) shows the degradation curves for the two types of samples under visible light irradiation. As shown in Fig. 6(A), the photocatalytic activities of samples are increased after annealing in nitrogen. The first order reaction kinetics of degradation of RhB under visible light is applied. The general pseudo-first-order model is shown as follows


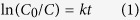


where *C*_*0*_ and *C* are the concentrations of the RhB dye in solution at time 0 and t, respectively, and *k* is the pseudo-first-order rate constant^45^. Figure 6(B) displays photocatalytic reaction kinetics of degradation RhB according to the data plotted in Fig. 6(A). It can be seen from Fig. 6(B) the RhB degradation rates are about 0.00803 and 0.05719 h^−1^ for the as-prepared samples and the samples annealed in nitrogen ambient, respectively. This indicates that the sample annealed in nitrogen shows a more than 7 times improvement in photocatalytic activity over that of the as-prepared sample. Moreover, compared with the commercial P25, the photocatalytic activity of the prepared BiLa_1.4_Ca_0.6_O_4.2_ annealed in nitrogen is significantly improved under visible light^46^. To better analyze the reasons for the improvement of the photocatalytic degradation rate, we measured the specific surface area (BET) of the samples by the static method. After annealing in nitrogen, the specific surface area increases from 2.39 m^2^/g to 6.74 m^2^/g which is improved by 2.82 times. Larger specific surface area make more photocatalytic reaction occur^46^.

From Fig. 6(A,B), it is found that the photocatalytic activities of the sample annealed in nitrogen is increased compared with that of the as-prepared sample. For the samples annealed in nitrogen, the absorption edge is blue-shifted and the band gap is increased (Fig. 4(B)). Meanwhile, the surface oxygen vacancy concentration is also increased (Fig. 5(A)). The broaden band gap would lead to less visible light asbsorbed. But, the introduction of defects as oxygen vacancy can boost the interfacial charge transfer, thus effectively reduce recombination rate of photo-generated electrons-holes^26^. Therefore, a net effect is that the photocatalytic activities of samples annealed in nitrogen are highly enhanced.

In summary, a new compound of BiLa_1.4_Ca_0.6_O_4.2_ is synthesized by solid state reaction, whose crystal structure is a rhombohedral R3mH structure with the hexagonal lattice constants of a = 3.893(1) Å, c = 9.891(1) Å. Its optical absorption edge is about 2.05 eV possessing an indirect band gap feature, which just spans the visible light region, validating it is a visible light sensitive photocatalyst. Its band gap is widened by oxygen vacancy produced from annealing in nitrogen. The photocatalytic activity of the BiLa_1.4_Ca_0.6_O_4.2_ powder to degradation of RhB is measured and improved more than 7 times by annealing in nitrogen ambient. The improved photocatalytic activity is ascribed to the increased oxygen vacancy concentration on surface of sample while annealing in nitrogen ambient. The current results suggest that further modification of BiLa_1.4_Ca_0.6_O_4.2_ by semiconductor coupling, noble metal decoration and nanocrystallization will doom improving its photocatalytic activity and providing new opportunities for solving the environmental pollution.

## Methods

### Preparation of BiLa_1.4_Ca_0.6_O_4.2_ samples

The BiLa_1.4_Ca_0.6_O_4.2_ samples were synthesized by solid state reaction of mixtures of Bi_2_O_3_, La_2_O_3_, and CaCO_3_ with the stoichiometric proportion. The mixed powders were ground in a mortar and pelletized in a grinding apparatus. Then it was preheated at 950 °C for 24 h in air followed by an intermediate grindings and pelletizing, and heated again at 1000 °C for 5 days. The samples were finally cooled to room temperature naturally. To modify the oxygen vacancy concentration in the BiLa_1.4_Ca_0.6_O_4.2_, and study its effect on photocatalytic activity, the prepared samples were annealed at 950 °C for 15 h in nitrogen flowing ambient. The flow rate of nitrogen was 50 mL/min.

### Characterization

The crystal structure of BiLa_1.4_Ca_0.6_O_4.2_ was analyzed by collecting powder X-Rar diffraction (XRD) patterns performed on a PANalytical Pert Pro X-ray diffractometer using Cu*K*a radiation at room temperature. The morphology of the samples was analyzed by a scanning electron microscopy (SEM) with a model Hitachi S-4800. UV-vis diffuse reflectance spectra of the samples were collected on Shimadzu UV-3600 Plus to reveal their band structure information. The core level spectra of oxygen atoms in the samples were collected in an X-ray photoelectron spectroscopy (XPS) of Pekin Elmer PHI-5300 XPS instrument to study the bonding configurations of the oxygen atoms. The Brunauer-Emmett-Teller (BET) specific surface area was measured with a V-Sorb 2800 apparatus.

### Photocatalytic activities

The photocatalytic experiments were carried out in a home-made photochemical Reactor equipped with a 300 W Xe lamp. To acquire visible light, a 420 nm cut off filter was applied between the lamp and the catalyst container. Rhodamine B (RhB) was adopted as the photocatalysis probe due to its high stability and sensitivity to visible light absorption due to its intrinsic absorption band at about 553 nm. In each photocatalytic experiment, 50 ml RhB aqueous solution in concentration 10 mg/L was filled into quartz beaker together with 50 mg BiLa_1.4_Ca_0.6_O_4.2_ powder. Before irradiation, the mixture was magnetically stirred in dark for 1.5 hours to approach adsorption-desorption equilibrium. The mixtures were then irradiated by visible light at room temperature and ambient pressure, while stirring to keep catalyst particles homogenously dispersed in solution.

During visible light irradiation, about 5 mL of the suspension was taken out from the beaker at a given time intervals about 2 hours in sequence for subsequent analysis of target dye concentration after centrifuging. Absorption spectra of the suspensions were collected by a Shimadzu UV-3600 Plus spectrometer. The photocatalytic activity of the BiLa_1.4_Ca_0.6_O_4.2_ was evaluated from the intrinsic absorption band (at 553 nm) intensity ratio of the remnant RhB after visible light illumination to that of the RhB in parent solution.

## Additional Information

**How to cite this article**: Zhong, W.W. *et al.* A new Bi-based visible-light-sensitive photocatalyst BiLa_1.4_Ca_0.6_O_4.2_: crystal structure, optical property and photocatalytic activity. *Sci. Rep.* **6**, 23235; doi: 10.1038/srep23235 (2016).
